# Peripheral cytotoxic immune profiles in hepatobiliary surgical patients

**DOI:** 10.3389/fimmu.2026.1861350

**Published:** 2026-06-03

**Authors:** Zhen Li, Dongping Yu, Lingyong Liu, Qi Zuo, Jiaqi Wang, Xiangjuan Jiao, Zhi Duan, Dong Chen

**Affiliations:** 1 Department of Pathology, The First Hospital of Changsha/The Affiliated Changsha Hospital of Xiangya School of Medicine, Central South University, Changsha, Hunan, China; 2 Department of Orthopedics, The Third Affiliated Hospital (the First Hospital of Nanchang), Jiangxi Medical College, Nanchang University, Nanchang, China; 3 Department of Emergency, The First Hospital of Changsha/The Affiliated Changsha Hospital of Xiangya School of Medicine, Central South University, Changsha, Hunan, China

**Keywords:** acute pancreatitis, cytotoxic lymphocytes, flow cytometry, granzyme B, immunosenescence, perforin

## Abstract

**Background:**

Perforin and granzyme B are key effector molecules of cytotoxic T lymphocytes (CTLs) and natural killer (NK) cells. Their expression profiles in hepatobiliary surgical patients and the relative contributions of disease-specific versus host factors remain unclear.

**Methods:**

In this retrospective cross-sectional study, 245 consecutive hepatobiliary surgical inpatients were enrolled. Six flow cytometric markers (CD3^+^, CD8^+^, and CD16^+^56^+^ subsets expressing perforin or granzyme B) were analyzed across six disease categories. Group comparisons were performed with and without age adjustment. Correlation, receiver operating characteristic (ROC), principal component analysis (PCA), and clustering analyses were conducted.

**Results:**

NK cells showed consistently high cytotoxic marker expression, exceeding T-cell subsets. No significant differences were observed across disease groups, even after age adjustment (all *p* > 0.05). All markers were positively correlated with age (ρ = 0.197–0.422; all *p* ≤ 0.002), indicating a dominant effect of immunosenescence. In acute pancreatitis (AP), CD8^+^Perforin^+^ was higher in severe cases and showed moderate discriminative performance [area under the curve (AUC) = 0.744; sensitivity 100%, specificity 50.0%]. CD8^+^Granzyme B^+^ correlated with bilirubin, albumin, AST, and lymphocyte count (all *p* ≤ 0.001). PCA identified two independent immune axes (T-cell and NK-cell cytotoxicity), and clustering revealed three immune phenotypes not associated with disease category.

**Conclusions:**

Peripheral cytotoxic marker expression is primarily driven by age rather than disease category in hepatobiliary surgical patients. CD8^+^Perforin^+^ shows potential as a marker for pancreatitis severity, while immune profiling reveals distinct but disease-independent cytotoxic phenotypes.

## 1 Introduction

Hepatobiliary and pancreatic diseases are a major cause of surgical admissions worldwide, encompassing conditions such as acute cholecystitis, choledocholithiasis, acute pancreatitis, and hepatobiliary malignancies (1). Despite advances in clinical management, complications including sepsis, organ dysfunction, and disease progression remain common, particularly in severe acute pancreatitis (SAP) and advanced malignancy (2–4). A shared feature across these conditions is disruption of host immunity. Inflammation, cholestasis, malnutrition, and tumor-associated immunosuppression all affect immune function, yet the cytotoxic immune response in hepatobiliary surgical patients has not been systematically characterized (5, 6).

Cytotoxic lymphocytes, including CD8^+^ T cells and NK cells, mediate cellular immunity through perforin and granzyme B (7). These molecules can be quantified by flow cytometry and serve as functional markers of cytotoxic activity. Although widely studied in oncology and infectious diseases, their role in common hepatobiliary conditions remains unclear (8). In AP, altered cytotoxic responses may contribute to disease severity, whereas in hepatobiliary and pancreatic malignancies and cholestatic conditions, immune dysfunction is a defining feature (9, 10). Age is a critical potential confounder. Immunosenescence alters cytotoxic lymphocyte profiles and may obscure disease-related signals in predominantly older patient populations (11). However, whether peripheral cytotoxic marker expression differs across disease categories, and what factors drive inter-individual variability, remains unknown.

In this study, we analyzed peripheral blood cytotoxic markers in 245 hepatobiliary surgical patients. We sought to determine whether cytotoxic marker expression differs across disease groups after accounting for age, to evaluate the relationship between age and cytotoxic activity, and to assess the value of CD8^+^ markers in distinguishing severe from mild acute pancreatitis (MAP). In addition, we explored the underlying immune structure using principal component analysis and unsupervised clustering.

## 2 Materials and methods

### 2.1 Study design and participants

This was a single-center, retrospective cross-sectional study. We included consecutive inpatients admitted to the Department of Hepatobiliary Surgery at the First Hospital of Changsha between June 2025 and January 2026. All patients underwent peripheral blood flow cytometry to assess cytotoxic lymphocyte markers. Inclusion criteria: patients were aged ≥16 years. Complete flow cytometry data for all six predefined markers were available. Exclusion criteria: patients had known primary immunodeficiency. Patients were receiving immunosuppressive therapy. Flow cytometry results did not meet internal quality control standards. The study was approved by the Ethics Committee of the First Hospital of Changsha (approval number: 2025-65). Written informed consent was obtained from all patients.

### 2.2 Disease classification

Patients were classified into six disease groups based on discharge diagnoses, namely pancreatitis (n = 57), benign biliary disease (n = 54), benign gallbladder disease (n = 50), hepatobiliary tumor (n = 42), benign liver disease (n = 25), and other conditions (n = 17). Benign gallbladder disease included acute cholecystitis and cholelithiasis with cholecystitis. Benign biliary tract disease included choledocholithiasis with cholangitis and obstructive jaundice. The pancreatitis group included MAP (n = 30), SAP (n = 9), and other pancreatic inflammatory conditions. Severe acute pancreatitis was defined according to the Revised Atlanta Classification (2012) as pancreatitis with persistent organ failure lasting more than 48 hours. Benign hepatic disease included non-alcoholic fatty liver disease and hepatic abscess. The tumor group included hepatocellular carcinoma, intrahepatic cholangiocarcinoma, pancreatic carcinoma, and other benign or malignant neoplasms. Benign disease categories were separated by anatomical origin because biliary, gallbladder, and hepatic pathologies involve distinct pathophysiological mechanisms that may differentially influence peripheral immune profiles. Malignant tumors were pooled into a single group because individual tumor subtypes had insufficient sample sizes for subgroup analysis, and all malignant conditions share systemic immunosuppressive effects on peripheral immunity.

### 2.3 Flow cytometric assessment of cytotoxic molecules

Peripheral blood samples were collected within 24 hours of hospital admission, prior to any surgical intervention or immunomodulatory treatment, to reflect the baseline immune status associated with each disease condition. Granzyme and perforin expression in CTLs and NK cells was quantified by multicolor flow cytometry using a commercially available granzyme/perforin detection kit (Qingdao Ruiskai Biological Technology Co., Ltd., Qingdao, China). All reagents were equilibrated to room temperature before use. The 10× lysis buffer was diluted 1:9 (v/v) with deionized water to obtain a 1× working solution. Two antibody cocktails were prepared: cocktail I contained anti-CD45, anti-CD3, anti-CD8, anti-CD16, and anti-CD56 antibodies, while cocktail II contained anti-granzyme and anti-perforin antibodies. For each sample, 25 μL of antibody cocktail I was pipetted into a flow cytometry tube, followed by the addition of 50 μL of whole blood. The mixture was vortexed for 3–5 seconds and incubated at room temperature (20–25 °C) in the dark for 15 min. Fixation was performed by adding 120 μL of fixative, followed by a further 15 min dark incubation. Cells were subsequently permeabilized by the addition of 2 mL of 1× permeabilization buffer, vortexed, and incubated in the dark for 15 min, then centrifuged at 200–300 × g for 5 min and the supernatant was discarded. The resulting pellet was labeled with 10 μL of antibody cocktail II for 15 min in the dark. Prior to acquisition, each tube was visually inspected for residual erythrocytes. Tubes with no visible red cell pellet were resuspended in 200 μL of 1× PBS; those with a visible pellet received 200 μL of 1× lysis buffer and were incubated for an additional 10 min before acquisition. Data acquisition was performed on a RaiseFlower flow cytometer (Qingdao Ruiskai Biological Technology Co., Ltd.). The antibody panel comprised: CD45-PE-Cy7, CD3-PerCP, CD8-APC-Cy7, CD16-APC, CD56-APC, anti-Perforin- FITC, and anti-Granzyme B-PE (all from Raisecare Biotechnology Co., Ltd., Qingdao, China). Lymphocytes were identified by CD45/SSC gating (3,000–5,000 events collected). CD3^+^ T cells, CD3^+^CD8^+^ T cells, and CD3^-^CD16^+^CD56^+^ NK cells were identified by sequential gating. Perforin and granzyme B expression was assessed within each respective parent population. A representative gating strategy is shown in **Supplementary Figure 1**. Before each run, sheath fluid levels and waste container capacity were verified, and fluidic initialization was completed following instrument warm-up. Routine maintenance, including fluidic cleaning, was carried out in accordance with the manufacturer’s recommendations to ensure system stability and data reproducibility.

### 2.4 Clinical and laboratory data collection

Baseline characteristics were retrieved from the electronic medical record system. These included sex, age, admission number, length of hospital stay, and discharge diagnosis. Laboratory data were collected concurrently. Complete blood count parameters included white blood cell count, absolute neutrophil count, absolute lymphocyte count, and lymphocyte percentage. Liver function tests included alanine aminotransferase (ALT), aspartate aminotransferase (AST), gamma-glutamyl transferase (GGT), alkaline phosphatase (ALP), direct bilirubin, indirect bilirubin, total protein, and albumin. Inflammatory markers included C-reactive protein (CRP) and procalcitonin (PCT). Coagulation parameters included D-dimer. For each analyte, the value closest to the date of flow cytometry sampling during the same admission was used.

### 2.5 Statistical analysis

All analyses were performed in Python (version 3.13.13) using standard scientific libraries, including SciPy, scikit-learn, and visualization packages. Continuous variables are presented as median [interquartile range (IQR)], and categorical variables as frequency (percentage). Normality was assessed using the Shapiro–Wilk test. As all flow cytometric markers were expressed as percentages, non-parametric tests were applied. Comparisons among the six disease groups were performed using the Kruskal–Wallis test. When appropriate, pairwise comparisons were conducted using the Mann–Whitney U test with Bonferroni correction. To account for the potential confounding effect of age, age-adjusted analyses were performed using a linear regression residual approach. For each marker, residuals from a regression model with age as the independent variable were used for subsequent group comparisons. Associations between flow cytometric markers and continuous variables were assessed using Spearman’s rank correlation coefficient (ρ). ROC curves and the AUC were used to evaluate the ability of each marker to distinguish severe from MAP. Optimal cut-off values were determined using the Youden index. PCA was applied after Z-score standardization to explore data structure. K-means clustering (k = 2–5) was used to identify potential immune phenotypes, with the optimal number of clusters determined by the silhouette coefficient. All tests were two-tailed, and *p* < 0.05 was considered statistically significant. To assess whether age was independently associated with cytotoxic marker expression after controlling for potential confounders, multivariable linear regression was performed for each marker with age, disease group (categorical), CRP, albumin, and lymphocyte count as covariates. Results are reported as regression coefficients (β) with 95% confidence intervals (CIs).

## 3 Results

### 3.1 Baseline characteristics

A total of 245 patients were included [137 males (55.9%)]. The median age was 59 years (IQR, 49–70), and the median length of hospital stay was 8 days (IQR, 6–13).

Patients were classified into six disease groups: pancreatitis (n = 57), benign biliary tract disease (n = 54), benign gallbladder disease (n = 50), tumors (n = 42), benign hepatic disease (n = 25), and other conditions (n = 17). The pancreatitis group was the youngest (median age, 39 years), whereas the benign biliary tract group was the oldest (median age, 66 years). Detailed baseline characteristics are presented in **Table 1**.

**Table 1:** Baseline characteristics of the study population (n = 245).

Characteristic	Value
Age, median (IQR), years	59 (49–70)
Age range, years	16–94
Male sex, n (%)	137 (55.9%)
Female sex, n (%)	108 (44.1%)
Length of stay, median (IQR), days	8 (6–13)
Disease group, n (%)	
Pancreatitis	57 (23.3%)
Benign biliary disease	54 (22.0%)
Benign gallbladder disease	50 (20.4%)
Hepatobiliary tumor	42 (17.1%)
Benign hepatic disease	25 (10.2%)
Other	17 (6.9%)

### 3.2 Overall distribution of flow cytometric markers

**Figure 1:**
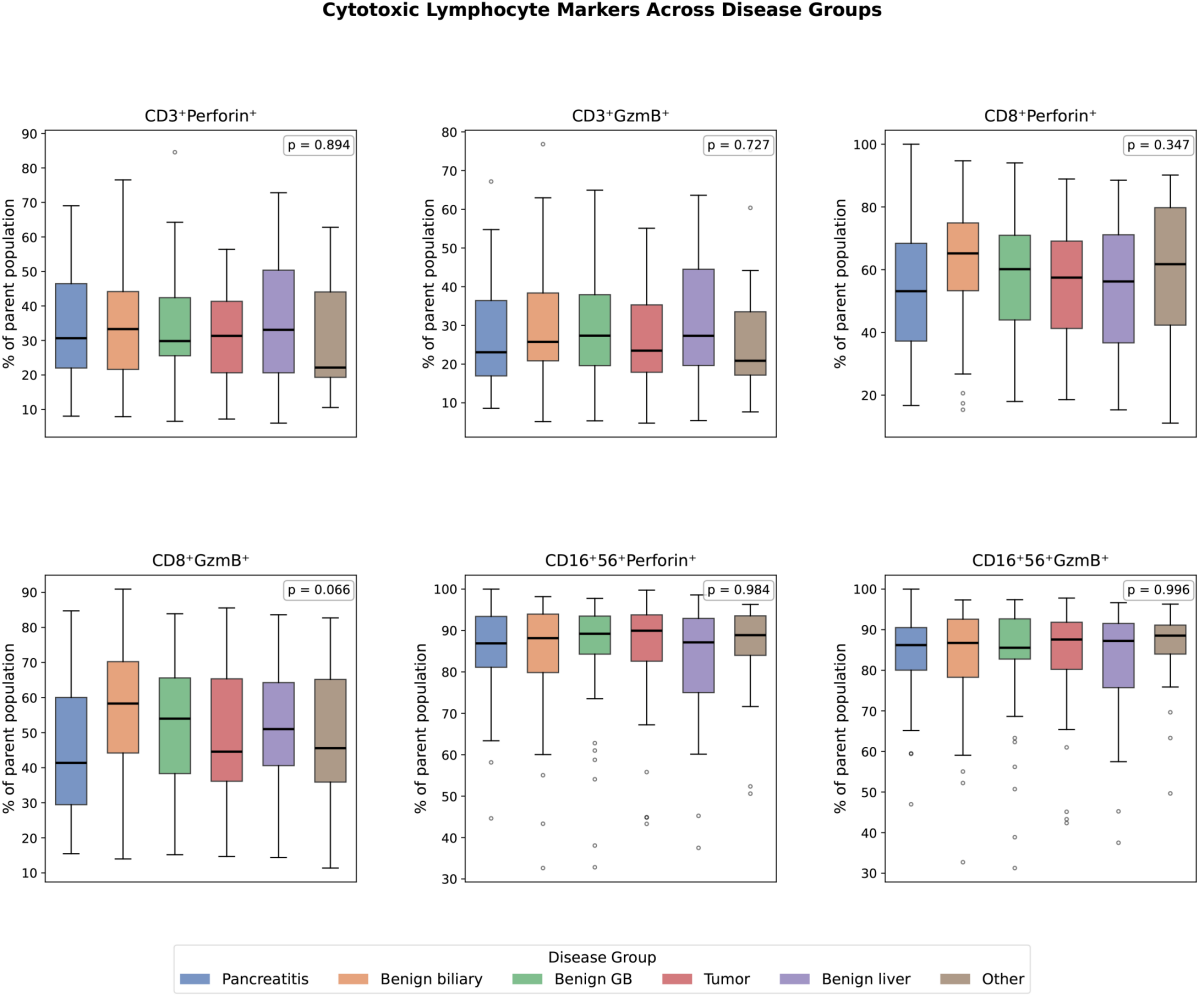
Distribution of cytotoxic lymphocyte markers across disease groups. Box plots of six flow cytometry markers across six hepatobiliary disease groups. Boxes indicate IQR, center lines indicate medians, and whiskers extend to 1.5×IQR. No significant differences were observed (all p > 0.05; GzmB+, Granzyme B+).

**Figure 2:**
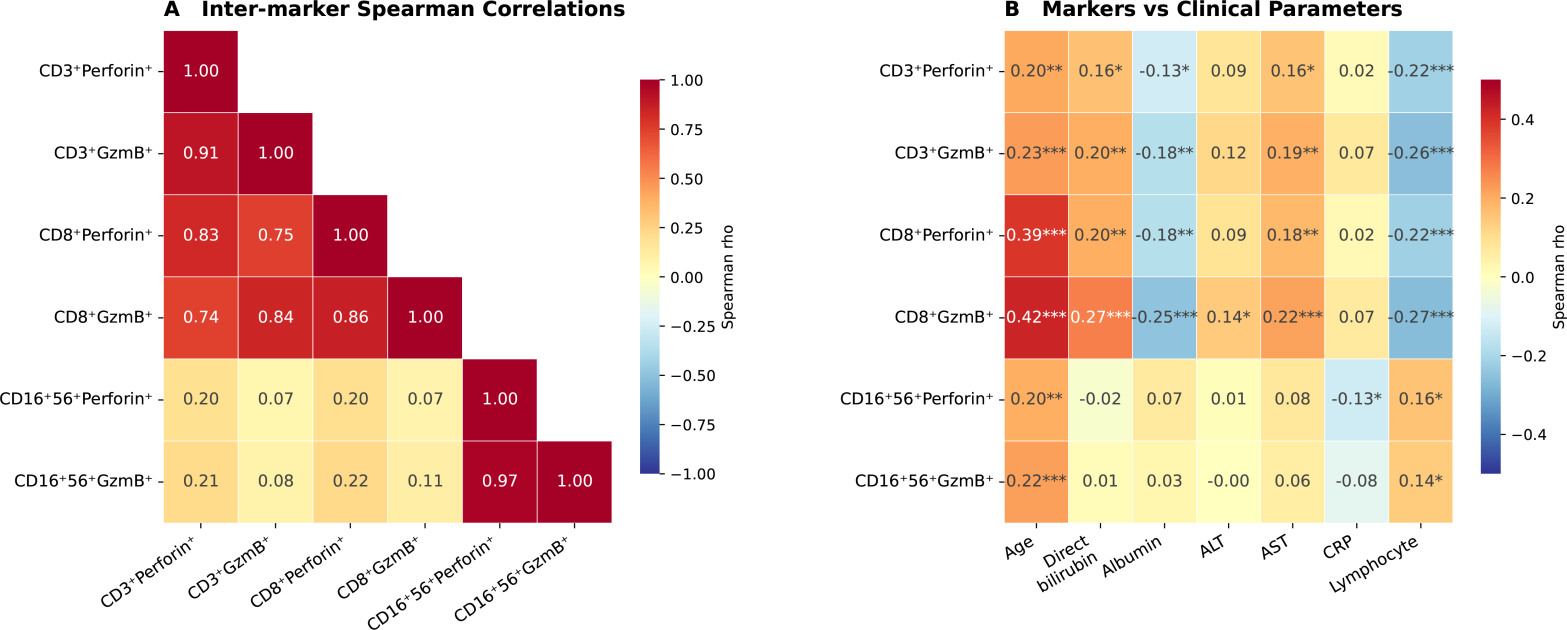
Correlation analysis of cytotoxic markers and clinical parameters. **(A)** Correlation matrix among six markers (all *p* < 0.001). **(B)** Correlations between markers and clinical variables. Asterisks indicate significance (*p < 0.05, **p < 0.01, ***p < 0.001).

Complete flow cytometric data were available for all 245 patients. Across the cohort, NK cells (CD16^+^56^+^) showed markedly higher positivity rates for cytotoxic molecules than T-cell subsets. The median CD16^+^56^+^Perforin^+^ was 88.2% (IQR, 81.4–93.6%), and CD16^+^56^+^Granzyme B^+^ was 86.9% (IQR, 79.5–91.9%). In contrast, CD3^+^ T-cell positivity rates were substantially lower. The median CD3^+^Perforin^+^ was 30.8% (IQR, 21.2–43.2%), and CD3^+^Granzyme B^+^ was 24.8% (IQR, 18.0–37.9%). CD8^+^ cytotoxic T lymphocytes showed intermediate levels. The median CD8^+^Perforin^+^ was 59.6% (IQR, 40.6–71.4%), and CD8^+^Granzyme B^+^ was 50.0% (IQR, 35.4–65.2%) (**Figure 1**). Within each cell subset, perforin and granzyme B were strongly correlated: CD3^+^ (ρ = 0.906), CD8^+^ (ρ = 0.864), and CD16^+^56^+^ (ρ = 0.970; all *p* < 0.001). In contrast, correlations between different cell types were minimal (CD8^+^ vs. CD16^+^56^+^: ρ = 0.07–0.22), suggesting independent regulation of cytotoxic activity in T cells and NK cells (**Figure 2A**).

### 3.3 Cytotoxic marker expression does not differ across disease groups

No significant differences in any of the six flow cytometric markers were observed across the six disease groups (all Kruskal–Wallis *p* > 0.05; **Table 2**). As age was identified as a potential confounder, analyses were repeated using age-adjusted residuals. The results remained non-significant for all markers (all *p* ≥ 0.355). Pairwise comparisons between tumor and non-tumor groups, as well as between the pancreatitis group and other groups, also showed no significant differences after Bonferroni correction. These findings indicate that peripheral blood cytotoxic marker expression does not distinguish among major hepatobiliary disease categories in this cohort.

**Table 2:** Peripheral blood cytotoxic lymphocyte markers across disease groups [median (IQR), %].

Marker	Pancreatitis (n=57)	Benign biliary (n=54)	Benign GB (n=50)	Tumor (n=42)	Benign liver (n=25)	Other (n=17)	*p*
CD3^+^ Perforin^+^	30.6 (22.0–46.5)	33.3 (21.6–44.2)	29.8 (25.6–42.4)	31.3 (20.6–41.3)	33.1 (20.6–50.4)	22.1 (19.3–44.1)	0.894
CD3^+^ GzmB^+^	23.1 (16.9–36.4)	25.7 (20.8–38.4)	27.3 (19.6–37.9)	23.5 (17.9–35.3)	27.3 (19.6–44.5)	20.9 (17.2–33.5)	0.727
CD8^+^ Perforin^+^	53.1 (37.2–68.4)	65.2 (53.3–74.9)	60.2 (44.0–71.0)	57.5 (41.3–69.1)	56.2 (36.7–71.1)	61.8 (42.3–79.8)	0.347
CD8^+^ GzmB^+^	41.4 (29.4–60.0)	58.3 (44.2–70.2)	54.0 (38.3–65.6)	44.6 (36.1–65.3)	51.0 (40.6–64.3)	45.6 (35.9–65.2)	0.066
CD16^+^56^+^Perforin^+^	86.9 (81.1–93.4)	88.2 (79.8–94.0)	89.2 (84.3–93.5)	89.9 (82.6–93.8)	87.1 (75.0–92.9)	88.9 (84.0–93.5)	0.984
CD16^+^56^+^GzmB^+^	86.2 (80.0–90.5)	86.7 (78.3–92.6)	85.5 (82.8–92.7)	87.6 (80.2–91.8)	87.2 (75.7–91.5)	88.5 (84.0–91.1)	0.996

KW, Kruskal-Wallis test; GzmB^+^, CD3^+^Granzyme B^+^; Benign GB, benign gallbladder disease.

### 3.4 Age is the primary determinant of cytotoxic molecule expression

**Figure 3:**
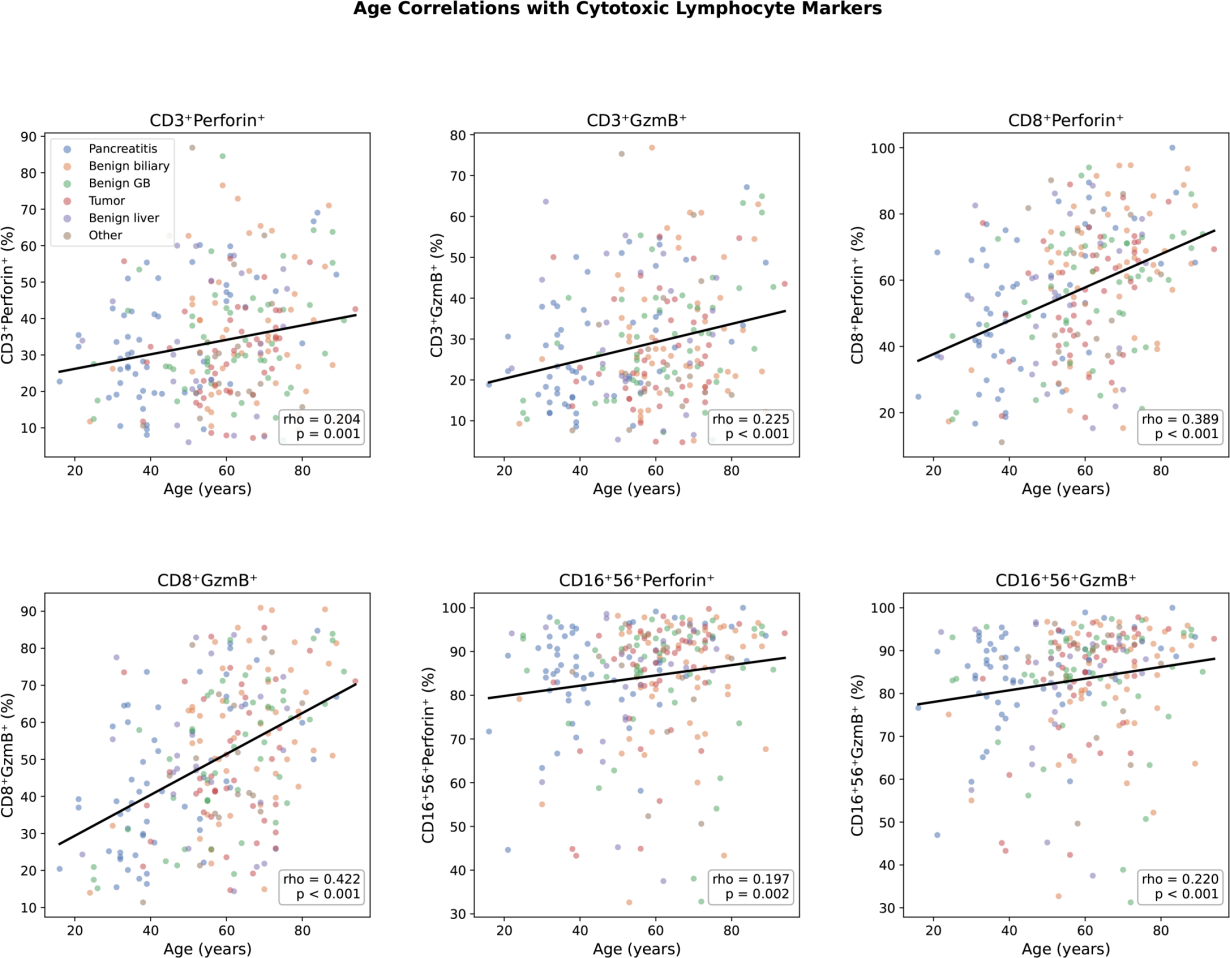
Age-associated changes in cytotoxic marker expression. Scatter plots showing correlations between age and cytotoxic markers. CD8^+^Granzyme B^+^ and CD8^+^Perforin^+^ showed the strongest associations (ρ = 0.422 and 0.389; both *p* < 0.001; GzmB+, Granzyme B+).

All six flow cytometric markers showed significant positive correlations with age (all *p* ≤ 0.002; **Figure 3**). The strongest associations were observed for CD8^+^ markers, including CD8^+^Granzyme B^+^ and CD8^+^Perforin^+^. CD3^+^ markers showed moderate correlations, whereas CD16^+^56^+^ NK-cell markers showed weaker but still significant associations. These results suggest that age is a major determinant of variability in cytotoxic marker expression, exceeding the influence of disease category in this cohort. To confirm that these associations were independent of disease category and inflammatory status, multivariable linear regression was performed adjusting for disease group, CRP, albumin, and lymphocyte count. Age remained a significant independent predictor for all six markers: CD3^+^Perforin^+^ (β = 0.177, 95%CI 0.011–0.343, *p* = 0.037), CD3^+^GzmB^+^ (β = 0.211, 95%CI 0.060–0.362, *p* = 0.007), CD8^+^Perforin^+^ (β = 0.485, 95%CI 0.295–0.674, *p* < 0.001), CD8^+^GzmB^+^ (β = 0.528, 95%CI 0.346–0.710, *p* < 0.001), NK Perforin^+^ (β = 0.235, 95%CI 0.102–0.368, *p* < 0.001), and NK GzmB^+^ (β = 0.248, 95%CI 0.118–0.378, *p* < 0.001). These findings confirm that the age effect is robust and not confounded by disease category, systemic inflammation, or nutritional status.

### 3.5 CD8^+^ Perforin^+^ is elevated in SAP

**Figure 4:**
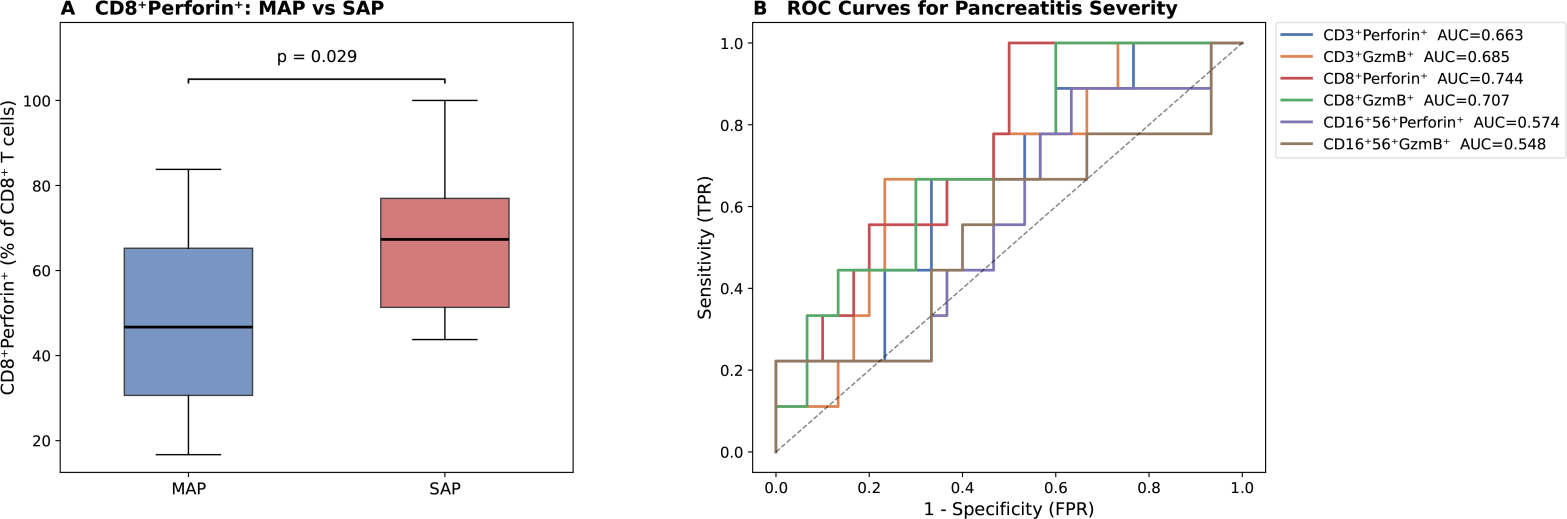
CD8^+^ cytotoxic marker expression in acute pancreatitis and ROC analysis. **(A)** CD8^+^Perforin^+^ expression in MAP and SAP. **(B)** ROC curves of six markers for distinguishing SAP from MAP. CD8^+^Perforin^+^ showed the highest performance (AUC = 0.744, 95%CI 0.561–0.899).

Among 39 patients with acute pancreatitis (MAP, n = 30; SAP, n = 9), CD8^+^Perforin^+^ was significantly higher in SAP than in MAP (**Figure 4A**). CD8^+^Granzyme B^+^ showed a similar but non-significant trend. No significant differences were observed for CD3^+^ or CD16^+^56^+^ markers. ROC analysis showed that CD8^+^Perforin^+^ had the best discriminative performance for SAP (AUC = 0.744, 95%CI 0.561–0.899). At the optimal cut-off of 43.8%, sensitivity was 100% and specificity was 50.0%, suggesting potential utility as a rule-out marker. Given the small SAP sample size (n = 9) and the wide confidence interval, these findings should be interpreted as exploratory and hypothesis-generating. The high sensitivity at the optimal cut-off may suggest potential utility as a rule-out marker, and clinical application should be approached with caution pending prospective validation in larger cohorts. Other markers showed lower performance, with CD8^+^Granzyme B^+^ as the next highest (AUC = 0.707), whereas NK-cell markers performed close to chance (**Figure 4B**). Detailed results are shown in **Table 3**.

**Table 3:** Comparison of cytotoxic markers between MAP and SAP patients.

Marker	MAP (n=30)	SAP (n=9)	*p*	AUC	Sensitivity	Specificity
CD3^+^Perforin^+^	31.5 (20.4–43.1)	40.9 (30.1–47.2)	0.147	0.663	67%	67%
CD3^+^GzmB^+^	22.6 (16.0–32.2)	33.8 (23.3–38.9)	0.099	0.685	67%	77%
CD8^+^Perforin^+^	46.7 (30.6–65.2)	67.3 (51.3–77.0)	0.029	0.744	100%	50%
CD8^+^GzmB^+^	40.3 (25.1–58.6)	52.2 (37.2–67.0)	0.064	0.707	100%	40%
CD16^+^56^+^Perforin^+^	87.2 (79.3–94.1)	87.8 (84.8–91.8)	0.516	0.574	—	—
CD16^+^56^+^GzmB^+^	85.6 (78.3–92.2)	88.7 (81.1–90.4)	0.677	0.548	—	—

Mann-Whitney U test. Optimal cutoff by Youden index.

### 3.6 CD8^+^Granzyme B^+^ correlates with markers of hepatobiliary dysfunction and lymphocyte depletion

**Figure 5:**
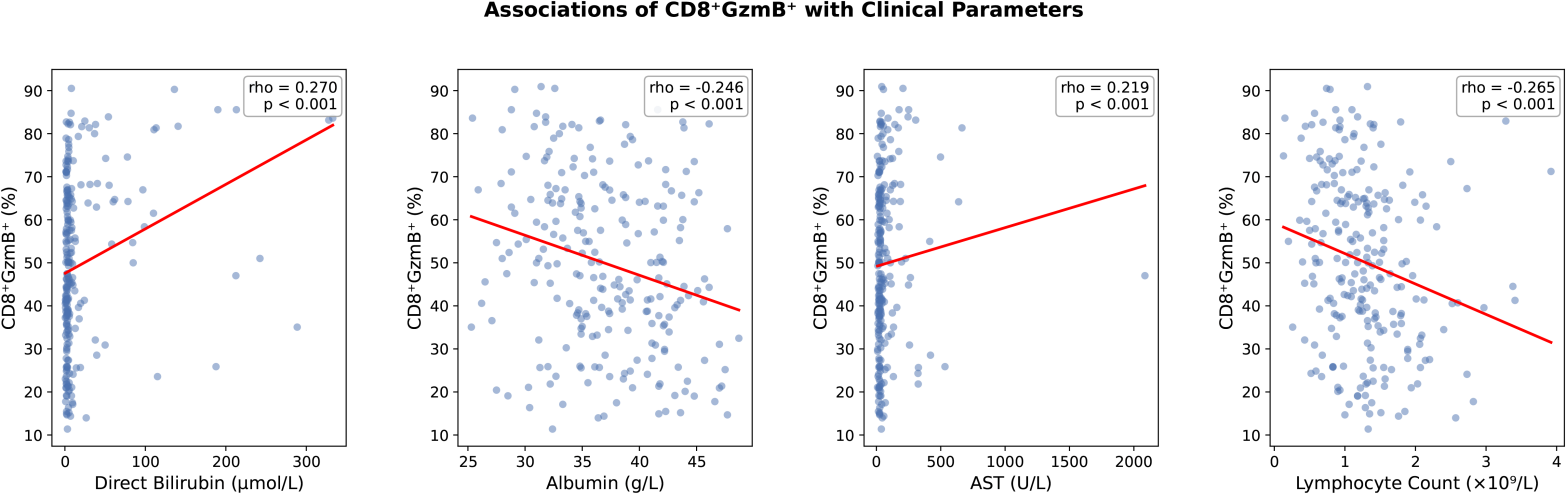
Associations between CD8^+^Granzyme B^+^ and clinical parameters. Scatter plots of CD8^+^Granzyme B^+^ versus direct bilirubin, albumin, AST, and lymphocyte count. Significant correlations are shown within each panel.

**Figure 2B** shows significant correlations between flow cytometric markers and clinical parameters. CD8^+^Granzyme B^+^ was associated with multiple laboratory variables, with the strongest correlations observed for direct bilirubin, absolute lymphocyte count, albumin, and AST. ALT showed a weaker but still significant association. A similar but less pronounced pattern was observed for CD8^+^Perforin^+^, which was also correlated with direct bilirubin, lymphocyte count, albumin, and AST. In contrast, CD16^+^56^+^Perforin^+^ showed only a weak inverse correlation with CRP. Overall, increased expression of CD8^+^ cytotoxic proteins (perforin and granzyme B) was associated with cholestasis, hypoalbuminemia, and lymphopenia (**Figure 5**).

### 3.7 Dimensionality reduction reveals two independent axes of cytotoxic function

**Figure 6:**
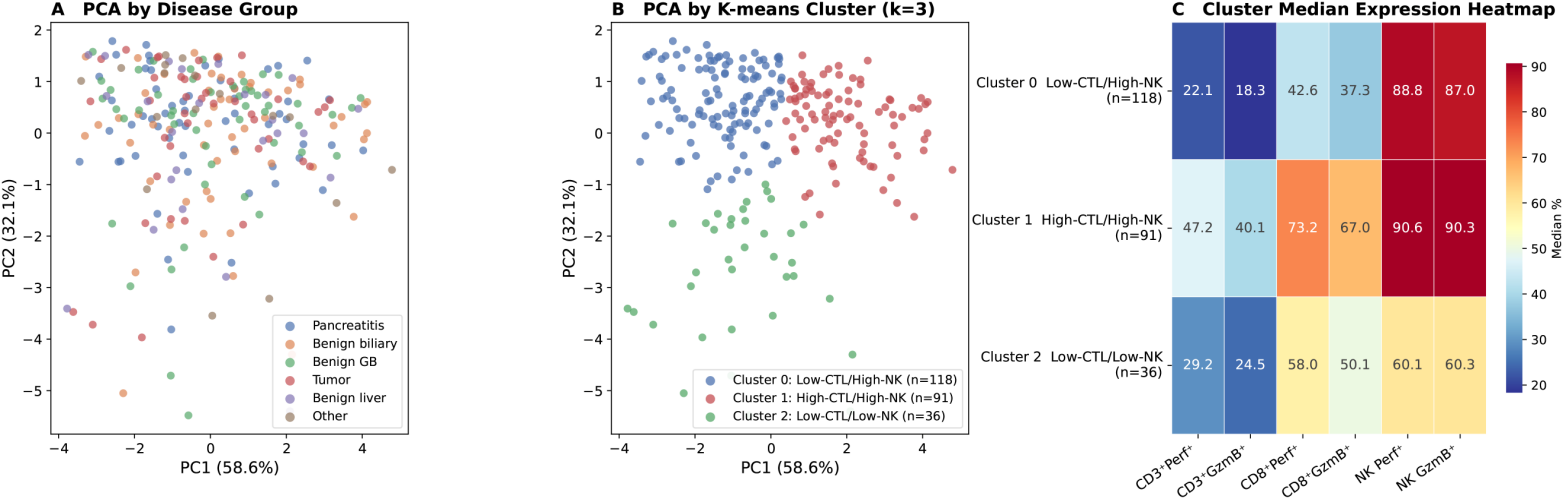
PCA and clustering of cytotoxic immune profiles. **(A)** PCA biplot of six markers colored by disease group. **(B)** PCA plot colored by K-means clusters (k = 3). **(C)** Heatmap of cluster-level marker expression showing three immune phenotypes (Low-CTL/High-NK, High-CTL/High-NK, Low-CTL/Low-NK; GzmB+, Granzyme B+; Perf^+^, Perforin^+^).

PCA of the six standardized markers identified two dominant components, explaining 90.7% of the total variance (**Figure 6**). PC1 accounted for 58.6% of the variance and was driven primarily by T-cell markers, with near-equal positive loadings (0.483–0.497) and minimal contributions from NK-cell markers (0.132–0.146). PC2 explained 32.1% of the variance and was dominated by NK-cell markers (CD16^+^56^+^Perforin^+^ and CD16^+^56^+^Granzyme B^+^; loadings 0.688–0.694), with negligible contributions from T-cell markers (−0.143 to −0.054). These results indicate that T-cell and NK-cell cytotoxic profiles represent two largely independent axes of variation in this cohort.

### 3.8 Unsupervised clustering identifies three immune phenotype clusters

K-means clustering identified three clusters (k = 3; silhouette coefficient = 0.398). Cluster 0 (Low-CTL/High-NK; n = 118) showed low T-cell cytotoxic activity with preserved NK-cell function. Cluster 1 (High-CTL/High-NK; n = 91) was characterized by uniformly high expression across all markers and had the highest median age (64.0 years). Cluster 2 (Low-CTL/Low-NK; n = 36) showed reduced NK-cell cytotoxicity with intermediate T-cell levels. No cluster was significantly associated with disease category (χ² p = 0.539). Detailed characteristics are shown in **Table 4**.

**Table 4:** Characteristics of three immune phenotype clusters identified by K-means clustering.

Variable	Cluster 0: low-CTL/high-NK (n=118)	Cluster 1: high-CTL/high-NK (n=91)	Cluster 2: low-CTL/low-NK (n=36)
n (%)	118 (48.2%)	91 (37.1%)	36 (14.7%)
Age, median (IQR), years	55.5 (39.5–65.8)	64.0 (54.0–74.0)	56.0 (45.8–70.2)
Male sex, n (%)	76 (64.4%)	44 (48.4%)	17 (47.2%)
Flow cytometry markers, median (IQR), %			
CD3^+^Perforin^+^	22.1 (17.0–29.5)	47.2 (41.0–56.8)	29.2 (23.1–35.0)
CD3^+^GzmB^+^	18.3 (14.1–22.1)	40.1 (33.7–51.5)	24.5 (20.6–32.4)
CD8^+^Perforin^+^	42.6 (33.4–55.3)	73.2 (67.7–81.7)	58.0 (42.8–66.6)
CD8^+^GzmB^+^	37.3 (24.9–44.4)	67.0 (61.3–75.3)	50.1 (38.0–61.3)
CD16^+^56^+^Perforin^+^	88.8 (84.0–93.5)	90.6 (86.4–94.6)	60.1 (45.2–67.2)
CD16^+^56^+^GzmB^+^	87.0 (82.8–91.7)	90.3 (85.1–93.7)	60.3 (49.0–65.6)
Disease group distribution, n (%)			
Pancreatitis	32 (27.1%)	21 (23.1%)	4 (11.1%)
Benign biliary disease	22 (18.6%)	22 (24.2%)	10 (27.8%)
Benign gallbladder disease	21 (17.8%)	22 (24.2%)	7 (19.4%)
Hepatobiliary tumor	21 (17.8%)	13 (14.3%)	8 (22.2%)
Benign hepatic disease	11 (9.3%)	9 (9.9%)	5 (13.9%)
Other	11 (9.3%)	4 (4.4%)	2 (5.6%)
χ² *p*-value (disease distribution)		*p* = 0.539	

GzmB, granzyme B.

## 4 Discussion

In this cross-sectional study of 245 hepatobiliary surgical inpatients, four main findings emerged. First, cytotoxic marker expression did not differ across major disease categories, even after adjustment for age. Second, age was the dominant determinant of variability, consistent with immunosenescence. Third, CD8^+^Perforin^+^ was elevated in SAP and demonstrated moderate discriminative performance. Fourth, dimensionality reduction and clustering analyses identified two independent immune axes and three phenotype clusters that were not associated with disease category. Taken together, these findings suggest that peripheral cytotoxic immunity is primarily driven by host factors rather than disease classification.

The absence of differences in perforin and granzyme B expression across disease groups is a clinically informative negative finding. Peripheral blood reflects systemic rather than tissue-resident immunity, and disease-specific cytotoxic responses are likely localized within affected organs rather than detectable in circulation. As such, circulating lymphocyte profiles may represent an integrated host immune state shaped by multiple systemic influences (12). In addition, the disease categories examined share a common feature of hepatobiliary or pancreatic stress, often accompanied by systemic inflammation. This shared background may lead to convergent peripheral immune profiles that obscure condition-specific differences. Similar convergence of peripheral T-cell phenotypes has been described in other inflammatory and surgical settings (13). From a clinical perspective, these findings suggest that peripheral perforin and granzyme B expression should not be used in isolation to distinguish among hepatobiliary disease categories. Instead, these markers appear to reflect host-level immune status rather than diagnosis.

All six cytotoxic markers were positively correlated with age, particularly within the CD8^+^ compartment, consistent with immunosenescence-associated accumulation of late-differentiated cytotoxic T cells. These cells constitutively express perforin and granzyme B, thereby increasing overall positivity rates with advancing age. The weaker age associations observed in NK-cell markers contrast with reports of declining NK-cell function in aging. This discrepancy may reflect shifts in NK-cell subset composition, with expansion of CD56^dim cytotoxic populations despite reduced functional capacity (14, 15). These findings highlight the distinction between cytotoxic molecule expression and actual effector function (16). The dominance of age as a determinant underscores the necessity of age adjustment in studies of cytotoxic immunity (11, 17). Without appropriate adjustment, age-related variation may be misinterpreted as disease-specific immune differences (18).

The elevation of CD8^+^ Perforin^+^ in SAP represents the most clinically relevant finding (19). Acute pancreatitis involves systemic immune activation, and CD8^+^ T-cell–mediated cytotoxicity has been implicated in tissue injury and organ dysfunction (20). The observed increase in circulating perforin-positive CD8^+^ cells is consistent with systemic CTLs activation in severe disease. CD8^+^ Perforin^+^ demonstrated moderate discriminative performance (AUC = 0.744), with high sensitivity but limited specificity. While insufficient as a standalone diagnostic tool, this profile suggests potential utility as a rule-out marker. Integration with established severity scores may improve risk stratification, although prospective validation is required. The absence of corresponding differences in NK-cell markers suggests that CTL activation, rather than NK-cell cytotoxicity, may be more closely linked to pancreatitis severity in the peripheral compartment.

CD8^+^ Granzyme B^+^ was associated with direct bilirubin, albumin, AST, and lymphocyte count, indicating a relationship with hepatobiliary dysfunction and systemic nutritional status. These findings suggest that cytotoxic T-cell activation co-varies with metabolic and inflammatory stress. Cholestasis and hepatocellular injury may promote systemic immune activation, while hypoalbuminemia and lymphopenia reflect altered immune homeostasis (21). Together, these factors may favor expansion of cytotoxic T-cell subsets expressing granzyme B. These observations position CD8^+^ Granzyme B^+^ as a potential integrative marker of hepatobiliary–immune interactions, although its clinical utility requires further investigation.

Unsupervised clustering identified three immune phenotype patterns defined by varying levels of CTLs and NK-cell cytotoxicity. These clusters were not associated with disease categories, reinforcing the conclusion that immune variation is driven primarily by host factors (22). The high-CTL cluster may reflect age-related immune remodeling, whereas the low-NK cluster suggests potential impairment of NK-cell cytotoxic capacity in a subset of patients (23). The clinical implications of these phenotypes remain unclear and warrant prospective study.

This study benefits from consecutive patient inclusion, complete flow cytometric data, and comprehensive assessment across multiple lymphocyte subsets. The use of age-adjusted analyses and data-driven approaches, including PCA and clustering, strengthens the robustness of the findings. Several limitations should be considered. The retrospective, single-center design limits generalizability. The cross-sectional nature precludes assessment of temporal dynamics. The sample size for severe pancreatitis was small, and findings in this subgroup should be considered exploratory. In addition, flow cytometry measures cytotoxic molecule expression rather than functional activity. Finally, unmeasured confounders may have contributed to residual variability. Furthermore, this study did not include measurement of soluble inflammatory mediators such as interleukin-6 or tumor necrosis factor-α; future integration of cytokine profiling may provide a more comprehensive understanding of the interplay between cytotoxic immune responses and systemic inflammation. The subgroup analysis of pancreatitis severity was feasible because acute pancreatitis provides a clinically well-defined severity spectrum with standardized diagnostic criteria; other disease groups in this study lacked analogous severity stratification, precluding equivalent analyses.

## 5 Conclusions

Peripheral blood expression of perforin and granzyme B in CD3^+^, CD8^+^, and NK-cell populations did not differ across major hepatobiliary disease categories. Cytotoxic marker variability was primarily associated with age, consistent with the influence of immunosenescence. Within the pancreatitis subgroup, CD8^+^Perforin^+^ was significantly higher in SAP and showed moderate discriminative performance (AUC = 0.744), suggesting exploratory potential as a complementary marker for severity stratification, pending prospective validation in larger cohorts. CD8^+^ Granzyme B^+^ was associated with direct bilirubin, albumin, AST, and lymphocyte count, suggesting links with hepatobiliary dysfunction and nutritional status. Dimensionality reduction and clustering analyses identified two independent immune axes (T-cell and NK-cell cytotoxicity) and three immune phenotype clusters. The clinical relevance of these patterns requires further validation. Overall, these findings characterize peripheral cytotoxic immune profiles in hepatobiliary surgical patients and highlight CD8^+^ Perforin^+^ as a potentially informative marker, particularly in the context of acute pancreatitis.

## Data Availability

The raw data supporting the conclusions of this article will be made available by the authors, without undue reservation.

## Supplementary material

Supplementary Figure 1 Representative gating strategy for flow cytometric analysis of cytotoxic lymphocyte markers. Lymphocytes were identified within the CD45^+^ population by CD45/SSC gating. Sequential gating was applied to identify CD3^+^ T cells, CD3^+^ CD8^+^ T cells, and CD3^-^ CD16^+^ CD56^+^ NK cells. Perforin and granzyme B expression was assessed within each parent population.
