# Supplementary figures and images for: Peripheral cytotoxic immune profiles in hepatobiliary surgical patients
Source: Front Immunol. 2026 Jun 3;17:1861350. doi: 10.3389/fimmu.2026.1861350 (PMC13271926; doi:10.3389/fimmu.2026.1861350)

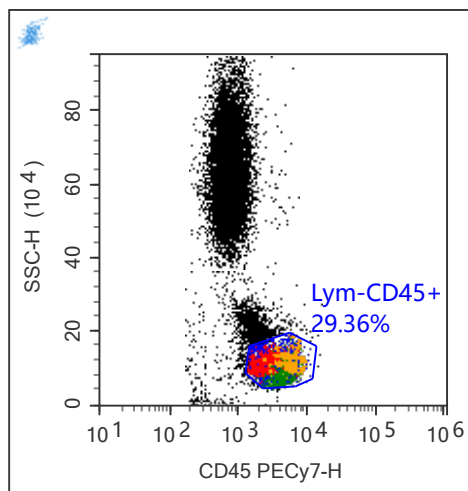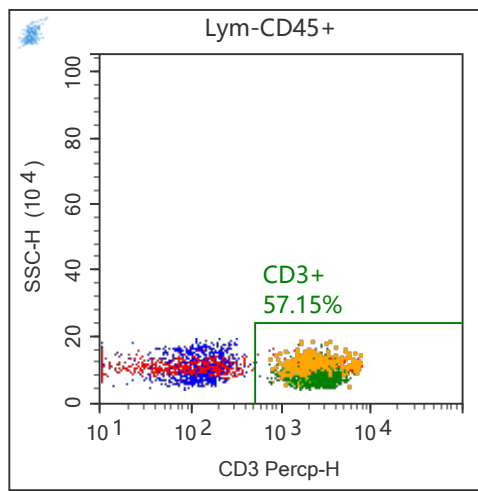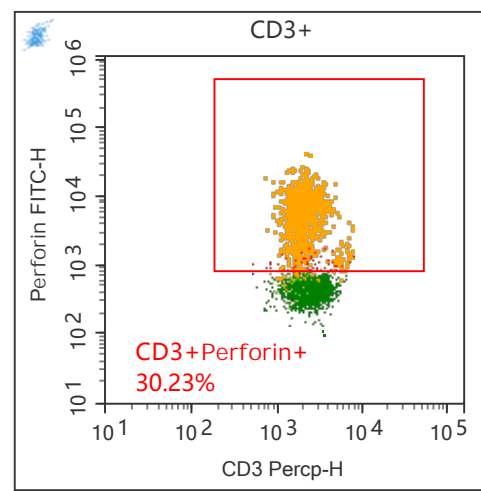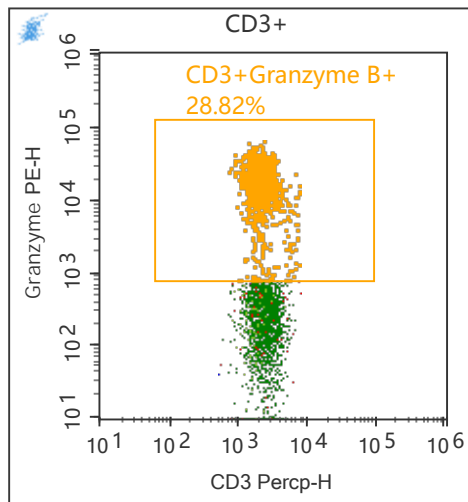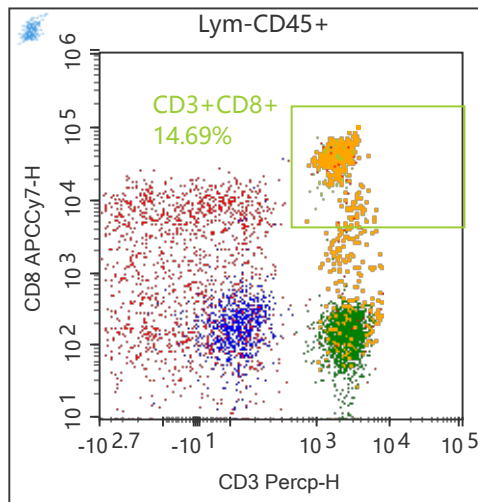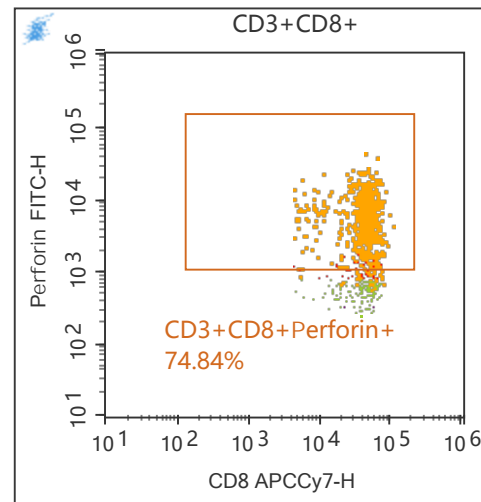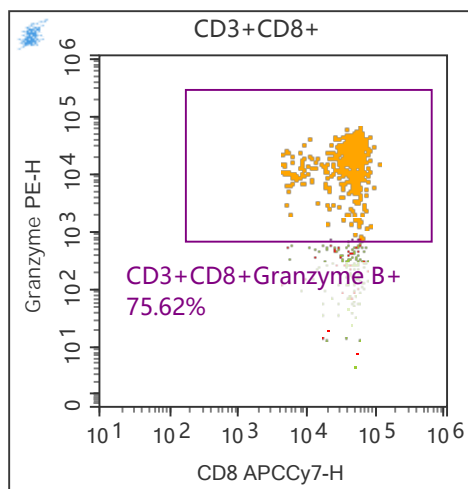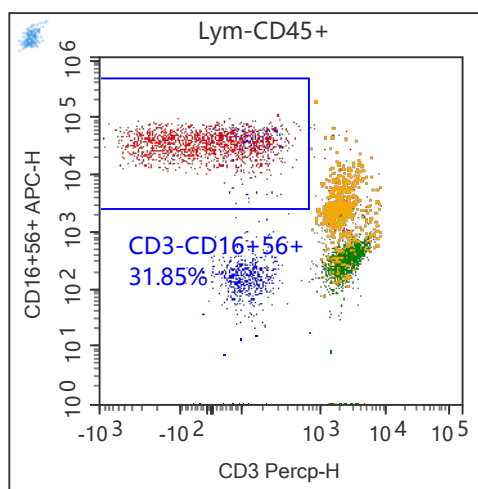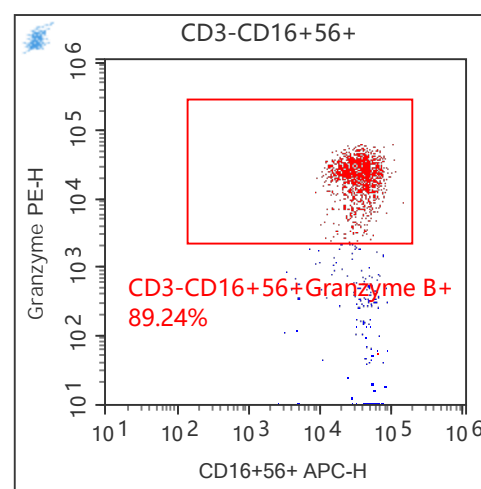

Supplement: Supplementary Figure 1 — Representative gating strategy for flow cytometric analysis of cytotoxic lymphocyte markers. Lymphocytes were identified within the CD45+ population by CD45/SSC gating. Sequential gating was applied to identify CD3+ T cells, CD3+ CD8+ T cells, and CD3- CD16+ CD56+ NK cells. Perforin and granzyme B expression was assessed within each parent population. [file Image1.pdf]
